# Evaluation of PD-L1 Expression and HPV Genotyping in Anal Squamous Cell Carcinoma

**DOI:** 10.3390/cancers12092516

**Published:** 2020-09-04

**Authors:** Anja Wessely, Markus V. Heppt, Claudia Kammerbauer, Theresa Steeb, Thomas Kirchner, Michael J. Flaig, Lars E. French, Carola Berking, Elisa Schmoeckel, Markus Reinholz

**Affiliations:** 1 Department of Dermatology, Universitätsklinikum Erlangen, Friedrich-Alexander-University Erlangen-Nürnberg (FAU), 91054 Erlangen, Germany; anja.wessely@uk-erlangen.de (A.W.); theresa.steeb@uk-erlangen.de (T.S.); carola.berking@uk-erlangen.de (C.B.); 2 Comprehensive Cancer Center Erlangen-European Metropolitan Area of Nuremberg (CCC ER-EMN), 91054 Erlangen, Germany; 3 Department of Dermatology and Allergy, University Hospital, LMU Munich, 80337 Munich, Germany; claudia.kammerbauer@med.uni-muenchen.de (C.K.); michael.flaig@med.uni-muenchen.de (M.J.F.); lars.french@med.uni-muenchen.de (L.E.F.); markus.reinholz@med.uni-muenchen.de (M.R.); 4 Institute of Pathology, Ludwig-Maximilians University (LMU), 80337 Munich, Germany; thomas.kirchner@med.uni-muenchen.de (T.K.); elisa.schmoeckel@med.uni-muenchen.de (E.S.)

**Keywords:** PD-L1, human papilloma virus, anal cancer, immune checkpoint, anal squamous cell carcinoma

## Abstract

**Simple Summary:**

Anal squamous cell carcinoma (SCC) is a rare cancer often caused by infection with high-risk human papillomavirus (HPV). Immune checkpoint blockade (ICB) targeting PD-1 or PD-L1 is successfully used for the treatment of other cancers as melanoma and lung cancer and might be an option for anal SCC as well. PD-L1 expression is associated with ICB outcome for example in melanoma. Here, we investigated whether infection with HPV affects PD-L1 expression and observed that almost two-thirds of anal SCC patients had PD-L1 positive tumors. Interestingly, these patients had a better median overall survival (OS). Sex, grade of differentiation, and HPV infection status did not influence the median OS. HPV infection status and PD-L1 expression were not correlated. Our findings indicate that PD-L1 is an independent prognostic marker in anal SCC. Besides, ICB targeting PD-1 or PD-L1 might be a therapy option for anal SCC patients irrespective of HPV infection status.

**Abstract:**

Anal squamous cell carcinoma (SCC) is a rare cancer with increasing incidence. Infection with high-risk human papillomavirus (HPV) subtypes is the major cause for its development. We retrospectively analyzed tumor samples from 54 anal SCC patients for infection with a panel of 32 HPV subtypes in a PCR-based approach, determined the PD-L1 expression status, and correlated the findings with the clinical data and the survival of the patients. Forty-two patients (77.8%) were HPV-positive and harbored at least one carcinogenic HPV subtype. HPV16 was the most frequently detected (*n* = 39, 72.2%). Four patients were infected with multiple HPV subtypes. HPV infection was significantly more often detected in female than in male patients (90.3% vs. 60.9%, *p* = 0.018). Patients with PD-L1 positive tumors showed a significantly better median overall survival (OS) compared with patients with PD-L1 negative tumors (69.3 vs. 28.3 months, *p* = 0.006). The median OS was significantly different among the distinct tumor stages (*p* = 0.029). Sex, grade of differentiation, and HPV infection status did not influence the median OS. Furthermore, HPV infection status and PD-L1 expression were not correlated. A multivariate Cox regression analysis revealed that PD-L1 expression status was an independent prognostic marker for survival (*p* = 0.012).

## 1. Introduction

Anal cancer is a rare cancer entity, affecting about 40,000 patients worldwide [1]. However, the incidence is steadily rising [2]. About 70 to 80% of the tumors are squamous cell carcinoma (SCC) [3], which can develop from precancerous lesions in the anal mucosa, termed anal intraepithelial neoplasia (AIN), with either mild (low-grade squamous intraepithelial lesion—LSIL) or moderate to severe (high-grade squamous intraepithelial lesion—HSIL) dysplasia [4]. Human papillomavirus (HPV) infections contribute to SCC development in different sites, including the cervix, oropharynx, and the anogenital tract [1]. A variety of HPV subtypes are found in more than 80% of anal cancer specimens, with HPV16 and HPV18 being the most commonly detected subtypes [1,3]. Furthermore, infection with human immunodeficiency virus (HIV), immunosuppression, smoking, a high number of sexual partners, and anal intercourse were identified as risk factors for anal cancer development [3,5]. 

Standard of care for most anal cancer patients is still a chemoradiotherapy (CRT) scheme, originally described by Nigro in the 1970s, that combines radiation therapy and chemotherapy with 5-fluorouracil and mitomycin [5]. However, CRT also causes a variety of short- and long-term side effects that may severely impair patients’ quality of life [6]. Furthermore, some patients do not respond to CRT or cannot be treated because of contraindications. Thus, new therapy options with less side effects are urgently needed.

The introduction of targeted therapies and immune checkpoint blockade (ICB) has tremendously changed the field of oncology in the last 10 years. ICB targets immune checkpoints, such as the CTLA-4 or the PD-1/PD-L1 axis with specific antibodies, and has achieved significant results in several cancer entities, including melanoma, non-small cell lung cancer (NSCLC), and squamous cell carcinoma of the head and neck (HNSCC) [7,8,9]. Recently, promising results have also been observed in a phase II trial investigating patients with metastatic or locally advanced cutaneous SCC who received the anti-PD-1 antibody cemiplimab [10,11]. Interestingly, anal SCC and HNSCC both arise from epithelial tissues and also share similar risk factors, including HPV infection and smoking [5,12]. Hence, ICB may be a promising candidate for anal SCC treatment as well [13]. However, ICB can also cause severe, mostly autoimmune-related adverse events [14]. Thus, patients should only receive ICB if they are likely to benefit from this treatment option. PD-L1 expression has been widely studied as a biomarker for predicting treatment response to ICB, and is associated with a better outcome in melanoma and NSCLC [15]. Yet, it is unclear whether infection with high-risk HPV types affects the expression of PD-L1 in anal SCC.

In this study, we analyzed tumor samples from 54 anal SCC patients for infection with a panel of 32 distinct HPV subtypes, determined the PD-L1 expression status on the tumor cells, and correlated the findings with patient survival in order to elucidate the relationship between infection with different HPV subtypes and PD-L1 expression in anal SCC.

## 2. Results

### 2.1. Baseline Characteristics

The baseline characteristics of the study population are summarized in Table 1. 

A total of 54 patients with anal SCC from stage I to stage IV were included in the study—31 of them were female (57.4%). Eight patients were categorized as stage I, 23 as stage II, 12 as stage III, and 11 as stage IV anal SCC. The median age at diagnosis was 60.1 years, ranging from 28.5 to 81.7 years. In most cases, the tumors were located at the anal canal and anal sphincter (ICD-10 code C21.1, 83.3%), followed by perianal skin (C44.55, 11.1%). The majority of tumors were poorly differentiated (51.9%).

**Figure 1:**
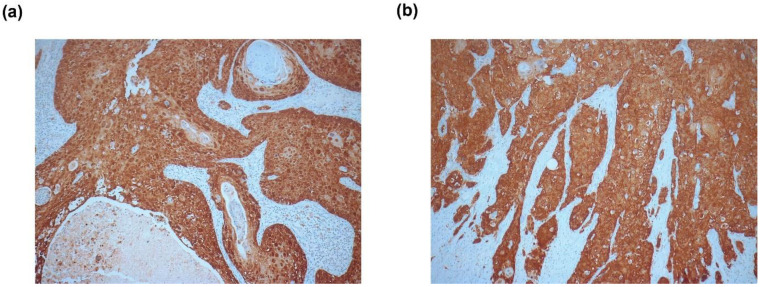
Representative images of positive p16 staining of two anal squamous cell carcinoma (SCC) samples. The tumors harbored (**a**) human papillomavirus-16 (HPV16) and (**b**) multiple HPV subtypes (HPV16 and HPV73), according to the PCR-based HPV array analysis. 100× magnification.

The viral oncoproteins E6 and E7 significantly contribute to HPV-induced carcinogenesis by deregulating the expression and function of several cell cycle-regulating proteins, including p53, pRb, and p16^INK4A^ (p16) [16]. Overexpression of the E7 protein in the transforming phase of the HPV infection increases the expression of p16 by altering its epigenetic regulation [17]. Hence, p16 is widely used as a surrogate marker for HPV infection [17,18]. In our patient cohort, p16 expression was present in 42 samples (77.8%) and absent in 11 samples (20.4%). The p16 expression status of one patient could not be evaluated. Representative images of immunohistochemical p16 staining are shown in Figure 1. Seven patients (13.6%) had a confirmed HIV infection and 36 (66.7%) were HIV negative; the HIV status for the other 11 study participants was unknown.

### 2.2. HPV Infection Status

**Figure 2:**
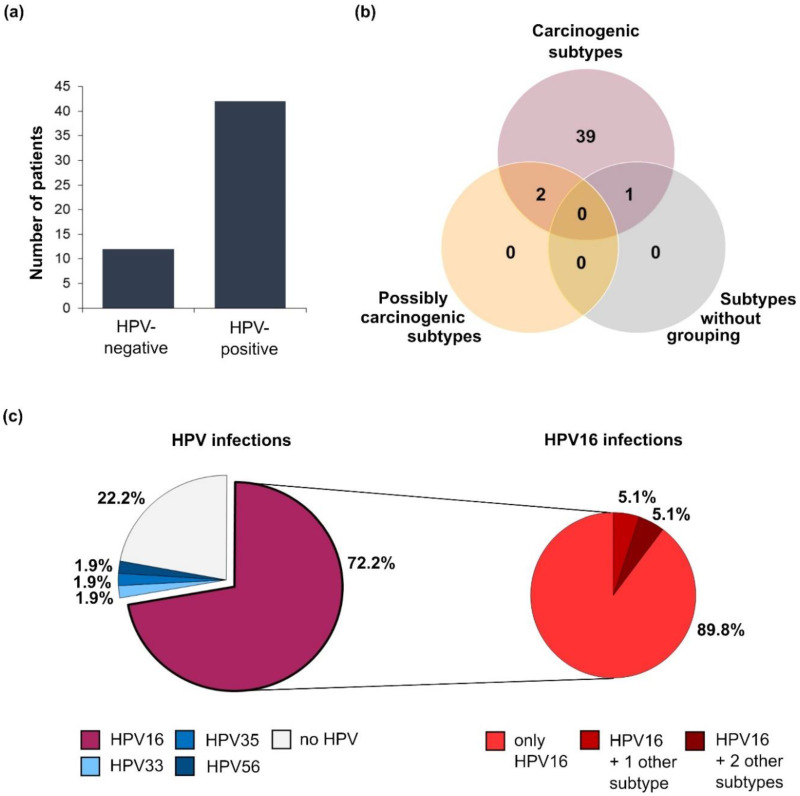
Distribution of HPV infections among the study participants (*n* = 54). (**a**) Bar chart illustrating the number of patients infected with different HPV subtypes; (**b**) number of HPV-positive patients harboring HPV subtypes categorized as carcinogenic, possibly carcinogenic, or not grouped according to the International Agency for Research on Cancer (IARC) classification; (**c**) pie charts showing the percentage of HPV infections grouped by subtype (**left**). About 10% of the patients harboring HPV16 were infected with multiple subtypes (**right**).

HPV DNA was detected in 42 out of 54 samples (77.8%, Figure 2). All HPV-positive tumors harbored subtypes, which were classified as carcinogenic by the International Agency for Research on Cancer (IARC) [19], namely HPV16, 18, 33, 35, and 56. HPV16 was present in 39 samples (72.2%), and thus by far the most frequently detected subtype. Four of the samples (7.4%) harbored multiple HPV subtypes (Table S1), and in one of these samples even three carcinogenic HPV subtypes (HPV16, 18, and 33) were detectable. All of the tumors with multiple HPV subtypes originated in the anal canal or sphincter (ICD-10 code C21.1). Interestingly, HPV-positive tumors were significantly more frequently detected in women than in men (90.3% vs. 60.9%, *p* = 0.018) and three of the four patients infected with multiple HPV subtypes were women.

### 2.3. PD-L1 Expression Status

**Figure 3:**
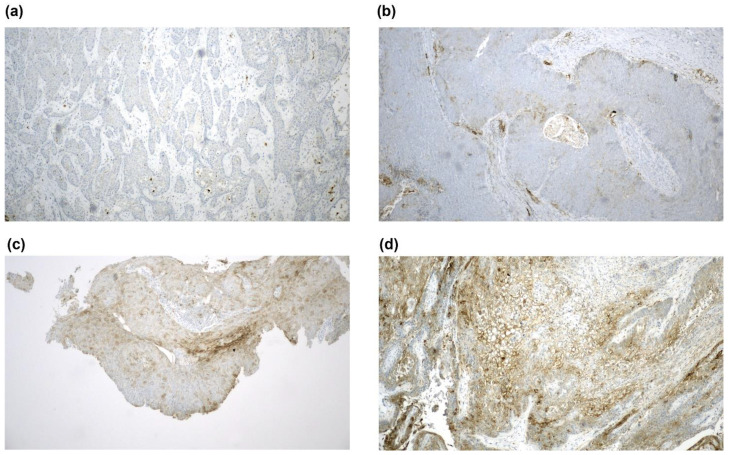
PD-L1 staining. Representative images of tumor samples categorized as (**a**) TPS category 0 (PD-L1 negative), (**b**) TPS category 1, (**c**) TPS category 3, and (**d**) TPS category 5. 100× magnification.

The PD-L1 expression status was evaluable in the tumor samples from 53 patients, and the staining intensity was evaluated and graded according to the tumor proportional score (TPS), as HPV is epitheliotropic (Table 2 and Figure 3).

The PD-L1 expression was detectable (TPS ≥ 1%) in 33 tumor samples (61.1%). An extremely high PD-L1 expression (TPS ≥ 50%, category 5) was observed in four samples (7.4 %). Notably, three of them were HPV-negative. In 20 samples (37.0%), no PD-L1 expression was detectable (TPS < 1%, category 0). Interestingly, a PD-L1 expression ≥ 10% (TPS category 3 and higher) was significantly more often detected in men (11/22, 50%) than in women (6/31, 19.4%; *p* = 0.019).

Comparing the HPV status and PD-L1 expression revealed that 27 out of 42 HPV-positive patients (64.3%), and 6 out of 11 HPV-negative patients (54.5%) showed an expression of PD-L1 (Table 3). The PD-L1 expression was only weak (TPS 1, *n* = 1) or even absent (TPS 0, *n* = 3) in the tumor samples of the four patients infected with multiple HPV subtypes. There was no significant difference of HPV infection status in tumors with vs. those without PD-L1 expression (*p* = 0.7285).

### 2.4. Survival Analysis

**Figure 4:**
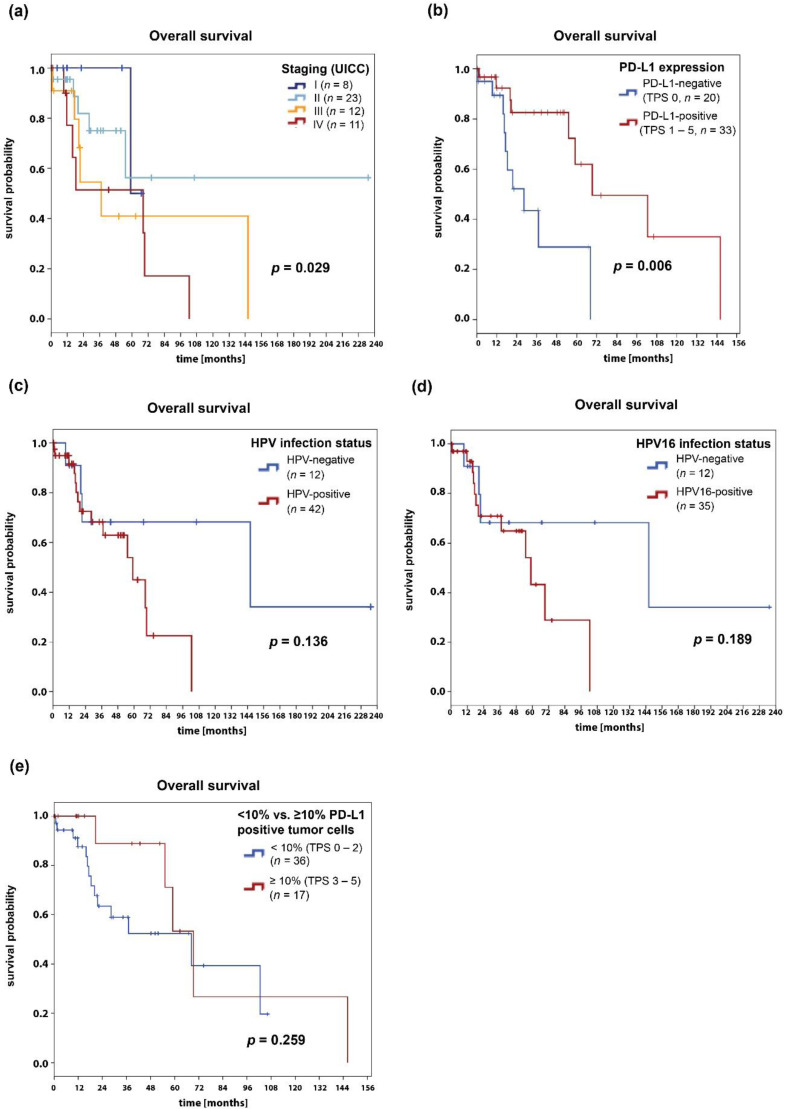
Survival analyses of the anal SCC patients. Kaplan–Meier curves show the overall survival depending on (**a**) tumor stage, (**b**) PD-L1 expression, (**c**) HPV infection status, (**d**) HPV16 infection status, and (**e**) PD-L1 expression (cut-off value: 10% PD-L1 positive tumor cells). *p*-values were calculated with the log-rank test and *p* < 0.05 were considered significant.

The median OS of all the patients was 68.3 months. The one-year, five-year, and ten-year OS rates were 91%, 55%, and 29%, respectively (Figure 4). The subgroup of patients with PD-L1 positive tumors (TPS 1–5) showed a significantly better survival compared with patients with PD-L1 negative tumors (69.3 vs. 28.3 months, *p* = 0.006). As expected, the median OS was significantly different among the distinct tumor stages (*p* = 0.029). Further subgroup analyses showed that the median OS was 68.3 months for men (*n* = 23) and 102.5 months for women (*n* = 31), but the differences were not statistically significant (*p* = 0.906). Additionally, no statistically significant survival differences were observed for good and intermediate vs. poorly differentiated tumors (*p* = 0.274). There was no difference of OS in patients with HPV-negative (*n* = 12) vs. those with HPV-positive (*n* = 42) tumors (median OS 146.1 vs. 59.0 months, respectively; *p* = 0.136). Neither was there an OS difference between patients with HPV16-positive and those with HPV-negative tumors (*p* = 0.189). Due to the small number of cases, we did not analyze the survival of patients infected with single vs. multiple HPV subtypes. Furthermore, there were no statistically significant differences in OS between patients with ≥10% PD-L1-expressing tumor cells (TPS category 3 or higher) and patients with <10% PD-L1-expressing tumor cells (TPS category 2 or lower, *p* = 0.259), between people living with HIV and HIV-negative patients (*p* = 0.714), and between immunosuppressed and immunocompetent patients (*p* = 0.948). 

In a multivariate cox regression model, PD-L1 expression was an independent risk factor and was associated with a favorable OS (PD-L1-negative vs. PD-L1-positive tumors: HR 0.27, 95% CI 0.10–0.75, *p* = 0.012). In this model, the tumor stage (HR 1.47, 95% CI 0.89–2.42, *p* = 0.134) and HPV16 infection status (HR 1.30, 95% CI 0.40–4.22, *p* = 0.663) were not associated with patient survival.

## 3. Discussion

PD-L1 expression on tumor cells has been widely investigated as a biomarker for predicting the outcome of ICB and as a prognostic marker. For example, NSCLC and melanoma patients with PD-L1-expressing tumors responded better to ICB targeting the PD-1/PD-L1 axis [15]. A subgroup of anal cancer patients expressed PD-L1 on the tumor cells, as reported in previous reports [20,21,22] and, interestingly, this was also the case in almost two-thirds of the patients in our study population, indicating that these tumors might be susceptible to ICB. Furthermore, most of the anal cancer patients were infected with HPV. Although infection with HPV is thought to initiate and drive anal cancer progression, HPV infection was associated with better local tumor control, PFS, and OS in a previous study [23]. HPV-positive tumors also showed a higher number of tumor-infiltrating lymphocytes (TILs), which is a favorable prognostic marker for CRT outcomes [24]. These findings indicate that HPV infection might attract TILs into the tumor bulks and trigger an anti-tumor immune response, leading to increased CRT efficacy. Antibodies blocking the interaction of PD-L1 on tumor cells and PD-1 on TILs may further enhance this response, as TILs already residing in the tumor remained functional, despite PD-L1 expression on tumor cells, likely resulting in a better and long-lasting tumor control.

ICB for anal cancer treatment is currently being investigated in several clinical trials. KEYNOTE-028 (NCT02054806), a multicenter open-label phase Ib study, investigated the safety and efficacy of the anti-PD-1 antibody pembrolizumab in various cancer entities, including anal SCC [13]. Patients with treatment-resistant locally advanced or metastatic disease and PD-L1 positive (≥1%) tumors were enrolled. Interestingly, 74% of all anal cancer patients that were screened for eligibility had PD-L1-positive tumors. This number of PD-L1 positive tumors was even higher than in our patient cohort (61.1%), indicating that PD-L1 expression is often observed in anal SCC. The antitumor efficacy of pembrolizumab was encouraging. The overall response rate of the subgroup of 24 anal SCC patients was 17% (95% CI 5.0–37%). Four patients had a partial response, and stable disease was observed in 10 out of 24 patients. The median PFS was 3 months and the median OS was 9.3 months. HPV infection status was only reported for three HPV-positive patients—two of them had a partial response and one patient did not respond [13]. Other promising results regarding the efficacy and toxicity have been reported in a single-arm, phase II trial (NCT02314169) investigating nivolumab, another PD-1 blocking antibody, in 37 patients with treatment-refractory metastatic anal SCC [25]. The authors observed that the tumor cells of the responding patients expressed significantly more PD-L1 than those of the non-responders. Notably, the HPV infection status was only determined in a subset of 15 tumors, which were all HPV-positive. Although the samples were tested for different HPV subtypes according to the study protocol, the frequency of these subtypes was not reported. Furthermore, the authors did not distinguish between HPV-positive and HPV-negative patients when reporting the outcomes, thus it remains to be elucidated whether HPV-positive tumors are more susceptible to ICB or not [25]. ICB has achieved the best results in cancer entities with a high tumor mutational burden, as these tumors are more likely to express neoantigens that can be recognized as foreign by immune cells [26]. Compared with other SCC entities such as NSCLC or cutaneous SCC, the mutational burden of anal SCC is not very high, with a median of 2.5 somatic mutations per megabase, which is comparable to those of other HPV-related cancer types such as HPV-positive HNSCC and cervical cancer [27,28]. Thus, the results of the ongoing trials with ICB in anal SCC have to be awaited.

PD-L1 was also examined in anal cancer, besides its putative role as a biomarker for predicting immunotherapy response. Evidence concerning PD-L1 expression and survival outcome for patients receiving CRT is controversial. Govindarajan et al. observed a poorer outcome of PD-L1 positive patients [20], and another study by Zhao et al. observed that PD-L1 positive patients had a poorer PFS and a trend towards poorer OS [22]. In contrast, patients with PD-L1 expressing tumors had a better local tumor control in the study published by Balermpas et al. [21]. Our findings are in line with the latter study, as patients with PD-L1 positive tumors had a prolonged survival compared with PD-L1 negative ones. Given the fact that our patients received stage-adapted standard of care treatment and none of them had ever received ICB, PD-L1 expression seemed to be an independent prognostic factor for survival, irrespective of the treatment option.

HPV infection is a major risk factor for developing anal SCC [1]. Our study confirms the results of previous reports showing that HPV can be detected in about 80 to 90% of tumors [1,3]. As expected, HPV16 was the most abundant subtype and was detected in more than 90% of HPV positive patients, and all HPV positive patients harbored at least one subtype categorized as carcinogenic by the IARC. A similar distribution of HPV subtypes was also observed by Morel et al. [29]. This recent study investigated the mechanistic signatures of HPV insertion in anal SCC. Episomal full-length HPV DNA was detected in 45% of the samples and, interestingly, the integration sites were evenly distributed throughout the human genome, except for one integration hotspot located near the NFIX gene on Chr. 19p13.2. Notably, HPV integration signatures and clinico-pathological features were not significantly associated. However, the authors did not evaluate the PD-L1 expression status of the patients [29].

It would be interesting to investigate if the survival of the patients and the PD-L1 expression of their tumors differed between tumors with different HPV subtypes. However, the number of the patients infected with subtypes other than HPV16 was too low for separate statistical analyses of the distinct subgroups. In our patient cohort, 90.3% of women, but only 60.9% of men, were infected with HPV. Interestingly, a small subgroup of four patients (7.4%) was infected with multiple HPV subtypes. This phenomenon has been previously observed in the cervical cells of HPV-positive women [30], but also in anal cancer patients, as shown by Lin et al. in their meta-analysis. Multiple infections seem to be correlated with HIV infection status, as 42% of HIV-positive patients, but only 10% of the HIV-negative patients were infected with more than one HPV subtype [31]. Unfortunately, we could not determine the HIV infection status of our subgroup members infected with multiple HPV subtypes. It also remains questionable whether an infection with multiple subtypes has an impact on survival. In our case, the number of patients, especially in the multiple HPV subgroup, was too small for a reliable survival analysis.

Except for the PD-L1 expression status, we did not find any significant correlations of sex, differentiation, or HPV infection status with the survival of the patients. This was quite surprising, as an improved OS for HPV-positive patients was observed in previous reports [23,32]. However, the sample size of this study was probably too was small to detect significant differences of survival between HPV-positive and HPV-negative patients. Furthermore, we observed no clear association between PD-L1 expression and HPV infection status in our patient cohort. This finding is in accordance with two other studies [20,22], suggesting that HPV infection status does not seem to have a huge impact on PD-L1 expression in anal SCC.

Vaccination against HPV might be able to boost the intrinsic anti-tumor response by reactivating immune cells to recognize and destroy tumor cells presenting viral antigens. Furthermore, it might also alter the expression of immune checkpoints on tumor cells or immune cells. Today, HPV infection with the two most common subtypes, HPV16 and HPV18, can be effectively prevented by the three commercially available vaccines, which cover distinct HPV subtypes that are associated with HPV-related cancers and the formation of genital warts [33,34,35]. However, these vaccines are only able to prevent, but not clear the infection. Instead, therapeutic vaccines aimed at generating a cell-based immune response instead of neutralizing antibodies have been developed and are currently being tested in several clinical trials [36]. However, no therapeutic vaccination approach has been licensed so far, and the trial results have to be awaited.

This study has some limitations. Anal cancer is a rare cancer entity and the availability of tumor material is sparse, thus we were not able to include a consecutive series of patients in this analysis. Furthermore, because of the exploratory nature of this study and the limited number of available samples, we did not perform a hypothesis-driven sample size calculation. The number of included patients was certainly small and the study may lack the power to detect significant differences, in particular regarding the subgroups of HPV-positive and HPV-negative patients. Furthermore, we could not conclude whether or not PD-L1 expression was linked to a response to immune checkpoint blockade, as the population had not been treated with immunotherapy. Nevertheless, it is possible that PD-L1 expression acts as prognostic factor, regardless of treatment with immune checkpoint blockers. Tumor stage was associated with the OS in the univariate analysis, but not in the multivariate Cox regression model. We assumed that this was due to the small number of patients in the stage subgroups and the uneven distribution of the patients.

## 4. Patients and Methods

### 4.1. Patient Population

The study was a retrospective analysis of 54 patients who were diagnosed and treated for anal SCC at the LMU University Hospital Munich between 15 February 1998 and 13 September 2017. Patients were included when archived tumor samples were available for further analyses. All of the patients received stage-adapted standard of care treatment, irrespective of HPV expression (surgery, radiotherapy, chemotherapy, or CRT). The clinical data and treatment outcomes were extracted from the patient records and were merged into a central database. The institutional review board of the medical faculty of the University Hospital approved the study (approval number 18-187 UE). Because of the explorative nature of this study, no power calculations were made in advance and the population was sampled for the availability of tissue specimens.

### 4.2. Analysis and Scoring of PD-L1 and p16 Expression

The PD-L1 and p16 expression of the tumor cells was determined by immunohistochemical staining in archived formalin-fixed paraffin-embedded (FFPE) tissue sections (*n* = 53). In one case, the PD-L1 and p16 expression analyses were not possible because of technical reasons. The sections were derived from surgical excisions, while biopsies were excluded and not assessed. Sections from entire tumor blocks were used to account for the intratumoral heterogeneity and variability of the PD-L1 expression. They were processed as described previously [37]. Briefly, sections were cut from paraffin blocks and mounted on SuperFrost Plus^®^ microscopy slides (Menzel Gläser, Braunschweig, Germany). Heat-mediated antigen retrieval was performed with a pressure cooker on a Ventana Benchmark XT autostainer (Ventana Medical Systems, Oro Valley, AZ, USA) with the XT UltraView diaminobenzidine kit (Vector Laboratories, Burlingame, CA, USA), and counterstained with hematoxylin (Vector Laboratories). Immunohistochemistry was performed for PD-L1 with clone SP263 (Ventana, ready-to-use) and for p16 with clone E6H4E6H4/p16INK4a (Ventana, ready-to-use). The percentage of PD-L1 positive cancer cells was independently evaluated by two trained dermato-pathologists (Markus V. Heppt and Markus Reinholz) and categorized with the tumor proportional score (TPS), as previously described by Scheel et al. [38], as we were specifically interested in staining the epithelial tumor cells. Only the membranous reactivity of carcinoma cells was considered and quantified relating to the amount of vital carcinoma cells. For further analysis and stratification, we applied scores referred to as TPS 0 (TPS < 1%), TPS 1 (1% ≤ TPS < 5%), TPS 2 (5% ≤ TPS < 10%), TPS 3 (10% ≤ TPS < 25%), TPS 4 (25% ≤ TPS < 50%), and TPS 5 (TPS ≥ 50%) [37]. Positivity for p16 was defined by strong cytoplasmic and nuclear staining throughout the whole tumor on a slide (“block” staining). Cases showing a weak or patchy staining were considered p16-negative. 

### 4.3. Identification of HPV Subtypes

FFPE tissue sections (10 µm, *n* = 54) were examined under an upright microscope and compared with the corresponding hematoxylin and eosin-stained sections. The representative tumor areas were marked manually by a trained dermato-pathologist (Markus V. Heppt). Subsequently, the sections were rehydrated with PCR-grade H_2_O, a 5 × 5 mm part of the tumor tissue was transferred into a tube containing 20 µL extraction buffer (100 mM Tris-HCl pH 8.0, 0.5 mM EDTA, 0.5% Tween 20, 0.9 mg/mL proteinase K) and was digested over night at 56°C. Afterwards, the samples were incubated for 10 min at 95 °C to inactivate proteinase K, and the HPV DNA was amplified using the polymerase chain reaction (PCR)-based LCD Array HPVType 3.5 (Chipron GmbH, Berlin, Germany), according to the manufacturer’s instructions with minor modifications. The PCR included an initial denaturation step (95 °C, 15 min), followed by 45 cycles (94 °C for 45 s, 45 °C for 1 min, and 72 °C for 1 min) and a final elongation step (72 °C, 10 min). Hybridizing of the amplified samples with the array plates was conducted according to the manufacturer’s instructions. For HPV subtype identification, the LCD array plates were scanned in a CHIP Scanner PF7250 (Chipron) and analyzed using SlideReader V12 Software (Chipron). The results were confirmed by examining the slides with a microscope at 40× magnification.

### 4.4. Statistics

Overall survival (OS) was defined as the time from initial diagnosis until death due to any cause. Survivors were censored at the time of the last documented follow-up. The survival probabilities were calculated with the Kaplan–Meier method and the survival curves were compared with the log-rank test. To investigate the relationship of the factors of interest with OS, a multivariate Cox proportional hazards regression model was calculated, as performed previously [39,40]. Hazard ratios (HR) with 95% confidence intervals (CI) were indicated to quantify the impact of a given factor on OS. Comparisons of variables with different groups were explored using *t*-test, Fisher’s exact test, or Chi-square test. Two-tailed *p*-values were calculated and two-sided *p*-values < 0.05 were considered significant. All of the analyses were carried out with SPSS statistics version 25.0 (IBM, Armonk, NY, USA).

## 5. Conclusions

In conclusion, our findings indicate that PD-L1 is expressed in both HPV-positive and -negative anal SCC, irrespective of the HPV subtype, indicating that ICB may be a promising treatment option for this patient group.

## Figures and Tables

**Table 1:** Baseline characteristics of the study population.

Patient Characteristics		Number of Patients (Unless Otherwise Stated)
Sex	Female	31 (57.4%)
	Male	23 (42.6%)
Age at diagnosis in years	Median (range)	60.1 (28.5–87.1)
	Mean (standard deviation)	58.9 (13.9)
Tumor location (ICD-10 code)	Anal canal, anal sphincter (C21.1)	45 (83.3%)
	Perianal skin (C44.55)	6 (11.1%)
	Overlapping lesion of rectum, anus, and anal canal (C21.8)	1 (1.9%)
	Unknown	2 (3.7%)
Grading	Poor differentiation	28 (51.9%)
	Intermediate differentiation	21 (38.9%)
	Good differentiation	5 (9.3%)
Staging	Stage I	8 (14.8%)
	Stage II	23 (42.6%)
	IIA	18 (33.3%)
	IIB	4 (7.4%)
	Stage III	12 (22.2%)
	IIIA	9 (16.7%)
	IIIC	3 (5.6%)
	Stage IV	11 (20.4%)
	Not classified	1 (1.9%)
HIV infection status	People living with HIV	7 (13.0%)
	negative	36 (66.7%)
	unknown	11 (20.4%)
Immune status	Immunocompetent	33 (61.1%)
	Immunosuppressed	10 (18.5%)
	People living with HIV	7 (13.0%)
	Multiple myeloma	2 (3.7%)
	Organ transplant recipients (kidney)	1 (1.9%)
	unknown	11 (20.4%)
p16 status	Positive	42 (77.8%)
	Negative	11 (20.4%)
	Unknown (not assessable)	1 (1.9%)

**Table 2:** Evaluation of the PD-L1 expression with the tumor proportional score (TPS).

TPS Category	PD-L1 Expression in Tumors	Number of Patients (%)
0	<1%	20 (37.0)
1	≥1% to <5%	10 (18.5)
2	≥5% to <10%	6 (11.1)
3	≥10% to <25%	7 (13.0)
4	≥25% to <50%	6 (11.1)
5	≥50%	4 (7.4)
-	Unknown/not assessable	1 (1.9)

**Table 3:** Distribution of HPV subtypes and corresponding PD-L1 expression status.

HPV Status	TPS						Total
	0 <1%	1 ≥1% to <5%	2 ≥5% to <10%	3 ≥10% to <25%	4 ≥25% to <50%	5 ≥50%	
**HPV-negative**	5	1	-	1	1	3	11
**HPV-positive**	15	9	6	6	5	1	42
• **HPV16**	11	7	5	6	5	1	35
• **HPV33**	-	-	1	-	-	-	1
• **HPV35**	1	-	-	-	-	-	1
• **HPV56**	-	1	-	-	-	-	1
**Multiple HPV**	3	1	-	-	-	-	4
**Total**	20	10	6	7	6	4	53

## Supplementary Materials

The following is available online at https://www.mdpi.com/2072-6694/12/9/2516/s1. Table S1: Patients infected with multiple HPV subtypes.
