# Supplementary material for: Evaluation of PD-L1 Expression and HPV Genotyping in Anal Squamous Cell Carcinoma
Source: Cancers (Basel). 2020 Sep 4;12(9):2516. doi: 10.3390/cancers12092516 (PMC7564961; doi:10.3390/cancers12092516)
Supplement: Supplementary file 1 [file cancers-12-02516-s001.pdf]

Supplementary Materials

Evaluation of PD-L1 expression and HPV genotyping in anal squamous cell carcinoma

Anja Wessely, Markus V. Heppt, Claudia Kammerbauer, Theresa Steeb, Thomas Kirchner, Michael J. Flaig, Lars E. French, Carola Berking, Elisa Schmoeckel and Markus Reinholz

Table S1. Patients infected with multiple human papillomavirus (HPV) subtypes. All of the patients were diagnosed with tumors located in the anal canal or sphincter (ICD-10 code C21.1).

Case no.	Sex	Number of detected HPV subtypes	HPV subtype category*		
			Carcinogenic	Possibly carcinogenic	No group
16	Female	2	HPV16	HPV73	-
22	Male	3	HPV16	-	HPV54, 90
35	Female	3	HPV16, 18, 33	-	-
39	Female	2	HPV16	HPV53	-

* according to the International Agency for Research on Cancer (IARC).

© 2020 by the authors. Licensee MDPI, Basel, Switzerland. This article is an open access article distributed under the terms and conditions of the Creative Commons Attribution (CC BY) license (http://creativecommons.org/licenses/by/4.0/).
